# Professor Robert Braidwood Sim—“Bob”—A Career in Complement Research Spanning 1973–2021

**DOI:** 10.3390/v13071190

**Published:** 2021-06-22

**Authors:** Kenneth Reid

**Affiliations:** Green Templeton College, University of Oxford, Woodstock Road, Oxford OX2 6HG, UK; kenneth.reid@bioch.ox.ac.uk

Bob joined the MRC Immunochemistry Unit, within the Department of Biochemistry at Oxford University, as a D.Phil. student in 1973, and quickly became immersed in the puzzle of the precise mechanism of the activation of the C1 complex. This involved isolating the proenzyme form C1 and then following its activation, via haemolytic assays in collaboration with Irma Gigli, a visiting USA scientist, originally from Argentina, who was already well established in research on the complement system. Irma, who brought South American flair to the Oxford lab, along with Bob, the quiet, but very confident, young Scotsman, made a good team and produced a sound paper about C1 activation, under the overall direction of Professor Rodney Porter [1]. The studies on the C1 complex, requiring the isolation of the unactivated C1 complex, and each of the subcomponents C1q, C1r, and C1s, illustrate how quickly, and expertly, Bob had mastered the techniques of protein chemistry [2], which he put to use, so well, all throughout his career. These two publications laid the groundwork for a major paper [3], again involving Bob, along with Alister Dodds, Mike Kerr, and Rodney Porter, in which it was proposed that “proenzyme C1r is activated autocatalytically, probably through the formation of an intermediary C1r*, in which the peptide chain in C1r* is unsplit, but a conformational change caused by interaction with the other components (within the C1q C1r_2_C1s_2_ complex interacting with IgG aggregates) led to formation of a catalytic site able to split, and thus activate, proenzyme subcomponent C1r, which in turn could then go on to activate proenzyme C1s”. It does seem that this concept is still relevant, after all these years, since the most recent studies on the precise mechanism of C1 activation, from several groups [4,5,6], indicate that this appears to remain a rather tricky and controversial research area, with no clear consensus view, despite the availability of excellent molecular models.

Bob was one of around six long serving senior scientists (Figure 1) who held posts in the Immunochemistry Unit and whose research, like Bob’s, was largely focussed upon the complement system. The Immunochemistry Unit’s research programmes were reviewed by the MRC every five years, and the senior scientists had to work closely together to ensure a good outcome for future funding. Bob was always a “safe pair of hands” during these stressful reviews by a visiting subcommittee, and all the other senior staff gratefully remember his sound and solid contributions to these occasions, which thankfully always ended successfully.

Bob’s influence on research carried out within the Unit is further emphasised by the appointment, in 1993, of one of his D.Phil. students, Tony Day, to the senior post vacated by Duncan Campbell (on Duncan’s appointment to an MRC Directorship in Cambridge). Tony Day, along with his student Simon Clark (Figure 2), have recently made great contributions to the understanding of the role that an allotypic variant seen in factor H may play in age-related macular degeneration [7,8,9]—this polymorphism being first described in research carried out in Bob’s lab [10].

There are many other areas, besides his research on C1 and factor H, where Bob’s far-reaching contributions (in over 340 publications) still impinge on concepts addressed in on-going complement research, which include the activation mechanisms of C3, as well as of MBL and MASPs; the characterisation of several regulators of the alternative and lectin pathways; IgG glycosylation in human health and disease; complement–pathogen interactions; and complement’s link with coagulation.

Bob continued to supervise students and contribute to research, via his visiting Professorship at Leicester University (in collaboration with Wilhelm Schwaeble) and contacts with Brunel University (in collaboration with Uday Kishore), and was one of the authors of a review [11] published in *Viruses* in 2021, thus marking over 45 years of Bob’s regular publications concerning the complement system.

Bob always paid great attention to the welfare of all the many D.Phil. students (over 23) and visiting research workers (over 30) who spent time in his Oxford laboratory, and ensured that they reached their full potential in their research, thus steadily building up an excellent network of collaborators as time progressed. He will certainly be sadly missed by everyone who interacted with him in the Immunochemistry Unit, and in his later positions, and who had experienced his generous approach in offering sound advice, and often essential reagents, to further their research in the complement field.

Our deepest condolences go to his wife Edith and all his family.

Ken Reid—on behalf of all ex-members of, and visiting scientists to, the Immunochemistry Unit, Oxford.

Professor Kenneth BM Reid, Emeritus Fellow, Green Templeton College, Oxford University, Woodstock Road, Oxford, OX2 6HG UK.

(Member of the Immunochemistry Unit 1969–2008, Director 1985–2008).

## Figures and Tables

**Figure 1:**
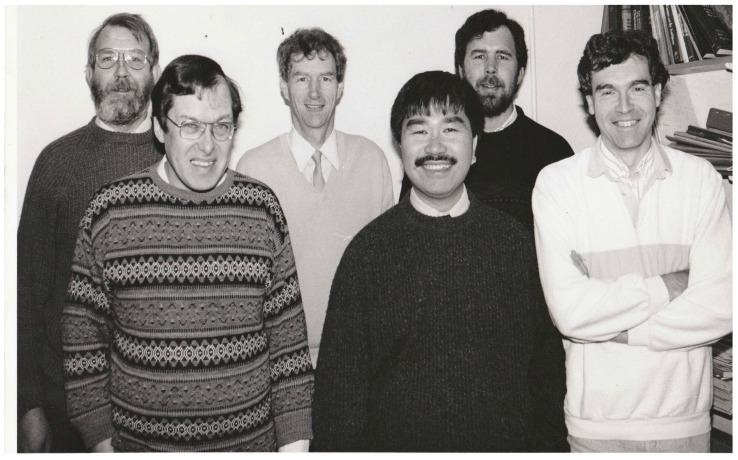
Front Row: Bob Sim, Alex Law, Duncan Campbell Back Row: Alister Dodds, Ken Reid, Tony Willis. In 1989, after one of the Immunochemistry Unit’s regular, and always successful, five year reviews of its research programmes, by the Medical Research Council.

**Figure 2:**
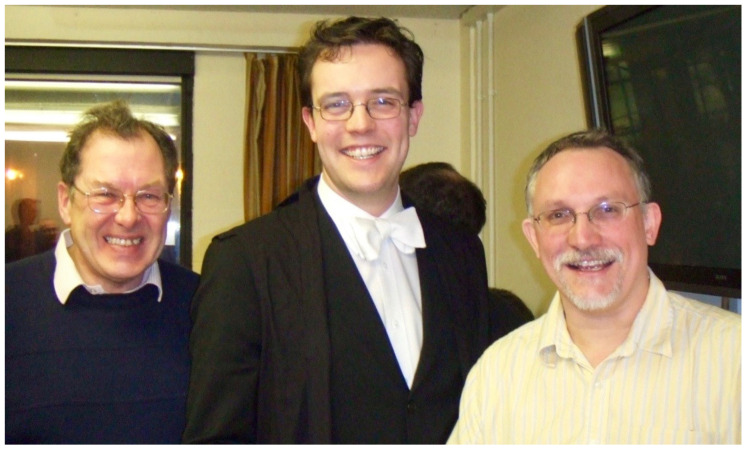
Bob Sim with Simon Clark and Tony Day. Taken after Simon’s DPhil viva in 2006. Tony now holds a Professorship at Manchester University and Simon holds a Professorship at Tuebingen University.
